# Water Impact of Syntactic Foams

**DOI:** 10.3390/ma10030224

**Published:** 2017-02-23

**Authors:** Adel Shams, Sam Zhao, Maurizio Porfiri

**Affiliations:** Department of Mechanical and Aerospace Engineering, New York University Tandon School of Engineering, Six MetroTech Center, Brooklyn, NY 11201, USA; adel.shams@nyu.edu (A.S.); sz1077@nyu.edu (S.Z.)

**Keywords:** fluid-structure interaction, hull slamming, hydroelasticity, particle image velocimetry, syntactic foams

## Abstract

Syntactic foams are particulate composite materials that are extensively integrated in naval and aerospace structures as core materials for sandwich panels. While several studies have demonstrated the potential of syntactic foams as energy absorbing materials in impact tests, our understanding of their response to water impact remains elusive. In this work, we attempt a first characterization of the behavior of a vinyl ester/glass syntactic subject to slamming. High-speed imaging is leveraged to elucidate the physics of water impact of syntactic foam wedges in a free-fall drop tower. From the images, we simultaneously measure the deformation of the wedge and the hydrodynamic loading, thereby clarifying the central role of fluid–structure interaction during water impact. We study two different impact heights and microballoon density to assess the role of impact energy and syntactic foam composition on the slamming response. Our results demonstrate that both these factors have a critical role on the slamming response of syntactic foams. Reducing the density of microballoons might help to reduce the severity of the hydrodynamic loading experienced by the wedge, but this comes at the expense of a larger deformation. Such a larger deformation could ultimately lead to failure for large drop heights. These experimental results offer compelling evidence for the role of hydroelastic coupling in the slamming response of syntactic foams.

## 1. Introduction

Syntactic foams are a class of lightweight composites that are synthesized by dispersing hollow particles in a matrix material [1]. The microballoon reinforcements provide a closed-cell microstructure, which in turn allows for a reduction in their structural weight [2,3], improving stiffness and strength [4,5], and controlling moisture absorption [6,7]. These characteristics have favored the integration of syntactic foams in marine vessels and aircrafts, as core materials for sandwich structures [8,9].

Several laboratory studies have demonstrated the potential of syntactic foams to absorb energy during impact across a number of experimental tests, including Izod impact (V-notched, U-notched, and unnotched), Charpy Impact, and drop weight. An excellent review of the state-of-the art can be found in [10], where experiments on vinyl ester, polyester, polypropylene, and polyvinylchloride matrix syntactic foams are comprehensively collated from the literature [11,12,13,14,15,16,17,18,19,20,21,22,23,24] and strengthened by new experiments on vinyl ester systems for varying microballoon wall thicknesses and volume fractions.

While these efforts have clarified the role of physical and microstructural properties on the impact response of syntactic foams, our comprehension of the behavior of syntactic foams as structural elements in marine vessels and aircraft remains elusive. A particularly challenging question entails the response of syntactic foams to slamming, which is routinely experienced by ship hulls during sailing and maneuvering [25] and seldom by airplanes during water landing and ditching [26]. Slamming is responsible for severe loading conditions, where pressure as high as few MegaPascal are attained in few milliseconds [27], thereby leading to undesired vibration and potentially failure [28]. In this work, we seek to bridge this gap by investigating the water impact of vinyl ester/glass syntactic foam panels.

Only a few experimental schemes have been proposed to study the slamming response of lightweight composites [29,30,31,32,33]. In particular, Charca et al. [29] and Charca and Shafiq [30] investigated the failure of polyurethane foam core sandwich panels under single and repeated slamming, and assessed the response of the composites for different impact energies and deadrise angles. Battley and Allen [31] developed an experimental setup based on a high-speed servo-hydraulic system to control the motion of an impacting hull fabricated from carbon fibre/epoxy skins sandwich composites. They measured the hydrodynamic loading on the hull and its structural response during slamming. Panciroli et al. [32] analyzed the free-fall water impact of fiberglass wedges, dissecting the role of hydroelastic phenomena through strain measurements and computer simulations based on smoothed particle hydrodynamics. Stenius et al. [33] further quantified the effect of hydroelastic phenomena in glass-fiber, glass-fiber/foam-core sandwich, and carbon-fiber/balsa-core sandwich panels impacting the water surface at a controlled speed. Currently, experimental data on the slamming response of syntactic foams are lacking. In this work, we analyze the influence of syntactic foam composition and impact energy on the slamming response of syntactic foam panels during free-fall impacts.

Different from any of the previous efforts on the slamming of lightweight composites [29,30,31,32,33], we propose the integration of particle image velocimetry (PIV) for the evaluation of the hydrodynamic loading experienced by the composites during the impact. PIV is an established technique in experimental fluid dynamics that is used to measure the velocity field in a particle-seeded fluid flow by correlating a sequence of high-speed images [34]. From the knowledge of the velocity field, one may reconstruct the pressure by integrating Navier–Stokes equations. Panciroli and Porfiri [35] and Nila et al. [36] first demonstrated the feasibility of using PIV in measuring the hydrodynamic loading experienced by rigid wedges during water impact. In contrast with the use of pressure sensors used in [29,30,31,33], PIV allows for a reconstruction of the hydrodynamic everywhere on the structure, without the need to instrument it. The knowledge of the hydrodynamic loading, coupled with high-speed imaging of the structural deformation, could be used to elucidate the physics of the impact and clarify the mechanics of failure.

Since its implementation in Panciroli and Porfiri [35], we have made considerable progress in demonstrating the accuracy of PIV-based pressure reconstruction and its potential to help explain hydroelastic phenomena during slamming. In two following studies, Panciroli and Porfiri [37,38] elucidated the role of structural flexibility on the response of metal structures. Specifically, in [37], they conducted experiments on a lightweight aluminum wedge at different impact heights, and utilized high-speed imaging to track deformations of the wedge during water entry. In [38], they investigated the feasibility of piezoelectric energy harvesting from the slamming of an aluminum wedge with a piezoelectric patch for different entry velocities.

In this work, we extend this methodology to the study of composite materials, toward elucidating the slamming response of vinyl ester matrix/glass microballoons syntactic foam panels. The panels were assembled in a wedge of 10${}^{\circ }$ deadrise angle, through a custom-made frame. Two microballoon densities were investigated to shed light on the role of syntactic foam composition on slamming response. Experiments were conducted in a drop tower, which allows for the control of the impact speed. Two drop heights were considered in the experiments to investigate the role of impact energy on slamming response. High-speed imaging was performed to measure the structural deformation of the panels and investigate the concurrent flow physics. These information were integrated with kinematic data of the wedge motion at its keel, obtained using an array of complementary sensors. Scanning electron microscopy (SEM) was utilized to study the fracture features of failed syntactic foam panels. Ultimately, experimental data allowed for a thorough characterization of the fluid–structure interaction, generated by water slamming of syntactic foams.

The paper is organized as follows. In Section 2, we describe our procedure for fabricating syntactic foams, the experimental setup used in our study, and the data acquisition system comprising the PIV apparatus and an array of built-in sensors. Therein, we also summarize our experimental conditions. In Section 3, we discuss our experimental observations on the physics of water impact for different types of syntactic foam panels and impact heights. We summarize the main conclusions of the study and present possible directions for future research in Section 4.

## 2. Experimental Methods

### 2.1. Fabrication of Syntactic Foam Panels

Syntactic foam panels used in the experiments were fabricated from glass microballoons and vinyl ester resin. Two types of glass microballoons (supplied by 3M (Saint Paul, MN, USA) [39]), with nominal densities of 220 and 460 kg/m${}^{3}$ (referred to S22 and K46, following the terminology of the manufacturer) were used to synthesize two syntactic foam compositions with inclusion volume fraction of 60%. These densities are often used as extreme cases of a “light” and “heavy” microballoon in experimental studies on polymer/glass systems [5,7,40]. We follow the same nomenclature used in [5] to identify the type of syntactic foams, such that the first number indicates the microballoon density and the second the volume fraction, both following “VE” for vinyl ester. As a result, we have two different types of syntactic foams: VE220-60 and VE460-60.

**Figure 1:**
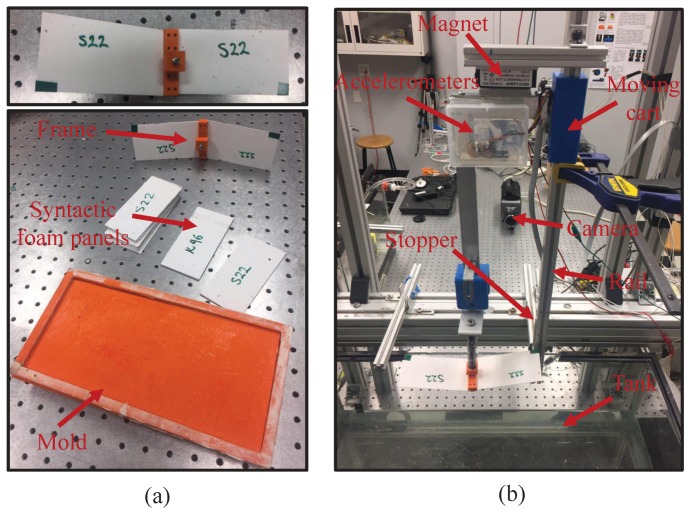
(**a**) In-house made mold for fabrication of thin syntactic foam panels, and custom-made frame to hold the panels during water impact; (**b**) Experimental setup with overlaid nomenclature of the components.

Four molds with dimension of 130 × 200 × 1 mm${}^{3}$ were made of acrylonitrile butadiene styrene material and printed in a Stratasys Dimension Elite rapid prototyping machine—see Figure 1a. We combined glass and vinyl ester resin based on their densities reported in [5]. Specifically, we first calculated the mass of the microballoons and vinyl ester with respect to the inclusion volume fraction and the volume of the mold. Then, we filled two separate beakers with the calculated amounts of microballoons and vinyl ester. Finally, we gradually poured vinyl ester into the beaker containing microballoons, while we mixed the mixture using a glass rod. After ten minutes of mixing, DEH 24 hardener of approximately 1% of the mass of vinyl ester was added to the mixture. The mixture was ready for molding once it was homogenized following another five minutes of mixing. The mixture was then gradually filled into the mold, and the excess was removed from the top of the mold to obtain a smooth top surface. The mold was rested on a flat surface for a day to fully harden. Composites were subsequently heated in a furnace for an hour at 100 ${}^{\circ }$. After the post-curing of matrix resin, the composites were ready for the machining process.

For machining, thin slices were milled off of the edges of the composites to obtain square corners. Then, each composite was milled into three smaller panels of in-plane dimensions 65 × 120 mm${}^{2}$. We sanded the thickness of the panels to achieve a target of 0.8 mm. The in-plane dimensions of the panels were selected to guarantee that the axial velocity had a secondary role on impact [41,42,43]. The thickness was chosen based on pilot simulations using the model proposed in [44], such that the peak stress during the impact would be in the range of experimental observations on syntactic foam strength [5,45]. The average densities (plus or minus one standard deviation) of the syntactic foam panels with S22 and K46 microballoons were 549.33±13.35 and 662.88±38.11 kg/m${}^{3}$, respectively. We calculated the relative difference between the density of the fabricated syntactic foam and the theoretical density to quantify undesired air porosity in the matrix material during fabrication [45]. The relative difference was 7.89% and 10.47% for S22 and K46 syntactic foam panels, respectively.

### 2.2. Experimental Setup and Data Analysis

We designed a frame to hold flat vinyl ester matrix/glass microballoons syntactic foam panels during impact using the Stratasys Dimension Elite rapid prototyping machine—see Figure 1a. The frame was designed to create a flexible wedge made of syntactic foams. A wedge-like geometry is in fact widely used in the literature to proxy hull slamming of a cross-section of the hull of a vessel [25]. The deadrise angle of the frame on each side was $10^{\circ }$, and the frame allowed the impact of syntactic foam panels with cantilever boundary condition.

Experiments were conducted in a drop tower apparatus controlled by a magnetic lock. At the top of the apparatus, the magnetic lock latched onto the wedge until the switch was turned off to release the wedge and initiate the free-fall impact. The wedge was guided by a moving cart sliding on a rail equipped with an SP-L-0750-203-3%-ST position sensor manufactured by Spectra Symbol to measure the moving cart’s displacement over time. A stopper was attached to the bottom of the rail to terminate the free-fall of the wedge once a sufficient penetration depth is reached. A water-resistant case with three accelerometers was attached to the moving cart to measure its acceleration. In particular, we used two 805M1 accelerometers with range of ±20 g and ±200 g, and one ADXL335 with range of ±3 g. The use of three separate accelerometers was intended to accurately resolve the motion of the wedge from the free-fall to slamming. A LabVIEW VI was programmed to keep the magnetic lock on, until a toggle button was pressed to release the wedge and initiate the data acquisition from the position sensor and the three accelerometers.

A transparent tank (800 mm long, 320 mm wide, and 350 mm deep) was suspended 80 cm above the floor by an aluminum frame. The tank was filled with water at room temperature and seeded with neutrally-buoyant polyamide particle tracers of diameter equal to 50 μm. A high-speed Phantom camera V.9.1 and a 5 W Nd:YAG Ray Power laser source were used in the PIV system—see Figure 1. The laser beam was reflected by a mirror mounted below the tank to illuminate the particles at the mid-span of the wedge. The camera was orthogonal to the laser, and the acquisition rate was set to 4 kHz. Due to the symmetry of the problem, only half of the wedge was visualized during the impact.

A moving mask was applied to PIV images to identify the boundaries of the wedge, water, and the air above the water surface. We extracted the velocity field from PIV images using the open source MATLAB code “PIVlab” [46]. The MATLAB code was based on a Fast Fourier transform cross-correlation algorithm and multi-grid scheme with a 50% interrogation window overlap, which utilized a decreasing interrogation area size algorithm with window sizes of 64 × 64, 32 × 32, and 16 × 16 pixels.

We employed our PIV-based pressure reconstruction technique [35] to calculate the pressure field in the fluid by integrating Navier–Stokes equations using a time marching integration method. Specifically, neglecting the effect of viscosity, gravity, and axial flow, the gradient of the pressure *p* can be expressed in terms of the measured velocity field through Navier–Stokes equations, as [47]:

(1a) $$\begin{array}{c} \frac{\partial p(x,y,t)}{\partial x}=-\rho_{w}\left(\frac{\partial u(x,y,t)}{\partial t}+u\left(x,y,t\right)\frac{\partial u(x,y,t)}{\partial x}+v\left(x,y,t\right)\frac{\partial u(x,y,t)}{\partial y}\right) \end{array}$$

(1b) $$\begin{array}{c} \frac{\partial p(x,y,t)}{\partial y}=-\rho_{w}\left(\frac{\partial v(x,y,t)}{\partial t}+u\left(x,y,t\right)\frac{\partial v(x,y,t)}{\partial x}+v\left(x,y,t\right)\frac{\partial v(x,y,t)}{\partial y}\right) \end{array}$$

where *x* and *y* are Cartesian coordinates in an inertial reference system; *t* is the time variable; $\rho_{w}$ is the water density, which was set to 1000 $\mathrm{kg}/m^{3}$, given that our experiments were at room temperature; and *u* and *v* are the velocity components along *x* and *y*, respectively, estimated from PIV data. We integrated Equation (1) in the entire fluid portion captured by the PIV image, imposing that the pressure on a point on the free surface is zero. By evaluating the pressure on the boundary of the wedge, we ultimately estimated the hydrodynamic loading on the syntactic foam panels. Specifically, the loading on the wedge was inferred from the pressure in the fluid at the closest point to the wedge.

Details of the implementation are presented in [35], along with a detailed comparison with classical results on rigid wedges. A critical assessment of the validity of the approach was conducted in [48], where synthetic data from computational fluid dynamics have been used to create a surrogate, uncertainty-free, dataset. Such a dataset was in turn utilized to evaluate the effect of the acquisition rate and spatial resolution on the accuracy of pressure reconstruction from PIV data. The feasibility of neglecting viscosity and gravity is discussed in [49,50], where predictions from Equation (1) were compared with findings obtained by retaining both effects in Navier–Stokes equations (the presence of viscosity in Equation (1) has a secondary role on the hydrodynamic loading [35]; in particular, with respect to the peak force, accounting for viscosity will cause variations of less than 2% [50]). Computational and experimental results addressing the role of the axial flow during water impact were presented in [41,42,48].

Experiments were conducted on two types of syntactic foams, VE220-60 and VE460-60, for two different drop heights, H=20 (short fall) and 40 cm (high fall), leading to four different experimental conditions: Light-High, Light-Short, Heavy-High, and Heavy-Short; see Table 1. The impact energy for the four experimental conditions was as follows: 1.37 J for Light-Short, 1.39 J for Heavy-Short, 2.75 J for Light-High, and 2.79 J for Heavy-High. Each experiment was repeated six times on six different samples for a total of 24 drop tests. To calculate the entry velocity, we integrated the ±3 g accelerometer data from the time when the magnet releases the wedge until the beginning of the impact. Table 1 shows the mean entry velocity $V_{0}$ with one standard deviation. We averaged the ±20 g accelerometer data over six trials for each condition and integrated the data from the beginning of the impact to obtain the time history of velocity. As the initial condition for the integration of the acceleration during the impact, we used the entry velocities reported in Table 1. The ±200 g accelerometer was used to ascertain the accuracy of acceleration data during the measurement, in case large accelerations are attained.

We utilized the “ginput” command in MATLAB to manually track in time the motion of the syntactic foam panels at the tip during the impact. For ease of tracking, we attached a marker at the tip of the panel.

After slamming experiments, we conducted SEM on dry syntactic foams using a Hitachi S-3400N SEM. Micrographs were taken on the fractured cross-section of the panels, coated with a layer of 5 nm of gold via a Sputter Coater 108 Auto-Set machine to minimize electron charging during microscopy.

## 3. Results and Discussion

Here, we analyze the response of two types of glass–vinyl ester syntactic foam with volume fraction of 60% entering the water surface at the two impact heights—20 and 40 cm. The role of hydroelastic phenomena on the syntactic foam panels can be estimated using the hydroelasticity factor. That is, $R_{F}=\frac{\tan\beta }{V_{0}}\sqrt{\frac{Eh^{3}}{\rho_{w}L^{3}}}$, where *E* is the effective Young’s modulus; $\rho_{w}$ is the density of the water; $V_{0}$ is the mean entry velocity; *β* is the deadrise angle of the wedge; and *L* and *h* are the length and thickness of the panel, respectively. Small values of $R_{F}$ are indicative of a strong hydroelastic coupling, with the structure undergoing large deformations during slamming [44]. Typically, the value of two is taken as a threshold for neglecting structural deformation during impact. We report the hydroelasticity factor for all the conditions in Table 1. The Young’s modulus is taken from the flexural modulus in [45]: E=2.34 GPa for VE220-60 and 3.75 GPa for VE460-60.

The first few natural frequencies of the panels can be computed from

(2) $$f_{i}=\frac{\lambda_{i}^{2}h}{2\pi L^{2}}\sqrt{\frac{E}{12\rho }},\;i=1,2,3,...\;,$$

where *ρ* is the syntactic foam density and $\lambda_{1}=1.8751$, $\lambda_{2}=4.694$, $\lambda_{3}=7.885$, and $\lambda_{4}=10.996$. For VE220-60, we have 20.174, 126.430, 354.006, and 693.711 Hz, and for VE460-60 we have 22.897, 143.493, 401.786, and 787.340 Hz. During water impact, added mass is expected to play a central role on the vibration of the panels [37,44,51], thereby leading to considerably slower oscillations compared to those associated with the natural frequencies of the “dry” panels in Equation (2).

### 3.1. High-Speed Imaging of the Impact

**Figure 2:**
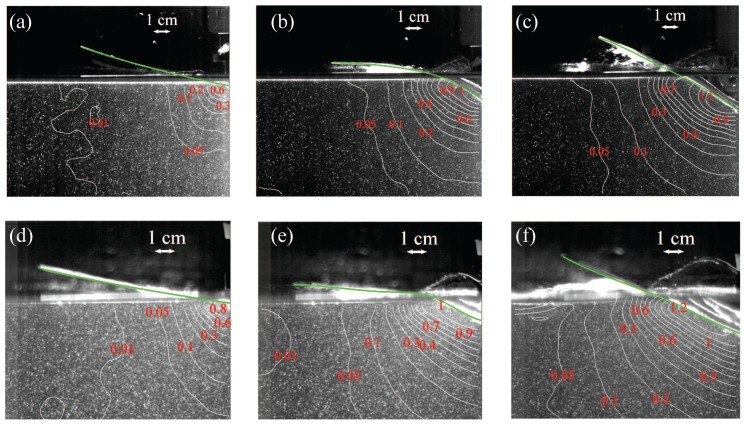
High-speed images overlaid with velocity magnitude in m/s for (**a**–**c**) Light-Short and (**d**–**f**) Heavy-Short conditions, at different time instants: (**a**,**d**) t=1.25 ms; (**b**,**e**) t=7.5 ms; and (**c**,**f**) t=12.5 ms. The green line identifies the panel shape.

**Figure 3:**
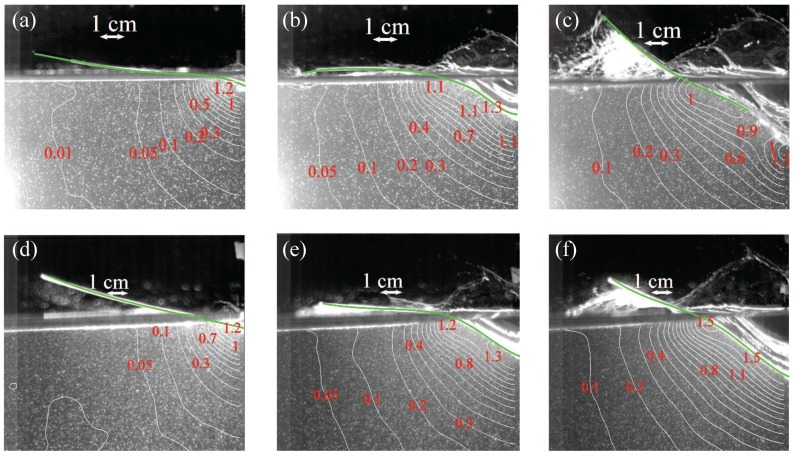
High-speed images overlaid with velocity magnitude in m/s for (**a**–**c**) Light-High and (**d**–**f**) Heavy-High conditions, at different time instants: (**a**,**d**) t=1.25 ms; (**b**,**e**) t=7.5 ms; and (**c**,**f**) t=12.5 ms. The green line identifies the panel shape.

To elucidate the effect of microballoon density and impact height on the velocity distribution and panel deformation during water entry, in Figure 2 and Figure 3 we overlay PIV images with velocity magnitude at t=1.25, 7.5, and 12.5 ms for all the experimental conditions. We also illustrate the deformation of the panels in Figure 2 and Figure 3 using a green line. For brevity, in Figure 2 and Figure 3 we only present results for one trial.

Figure 2 demonstrates that for an impact height of 20 cm, the velocity field during water entry of VE460-60 panels is larger than the velocity field for VE220-60 panels at the same time instants. This observation may be explained by the higher entry velocity of the heavier panel, which causes a larger energy to be imparted to the fluid flow. The velocity field increases at the keel for both wedges as time advances, different from results reported in [49] for the water entry of rigid wedges. The time evolutions of the structural deformation of VE220-60 and VE460-60 panels are approximately similar, whereby the panel vigorously bends downward as the wedge touches the water surface and then rises up as a wider length of the wedge is wetted during the slamming, in agreement with [37]. The initial downward bending is related to the inertia of the panel, which is contrasted by the deceleration at the keel. For both types of syntactic foam, the maximum downward deformation is attained at approximately between 5 and 6 ms; see Figure 2b,e and Table 1.

Similar to Figure 2, Figure 3 shows the velocity field and syntactic foam deformation for the impact height of 40 cm. Predictably, the velocity field and the structural deformations increase as the impact height increases from 20 to 40 cm [37]. By comparing the magnitude of the velocity field between Figure 2 and Figure 3, we observe a nearly 50% increase, which is associated with an analogous increase in the entry speed. A larger water splash is also evidenced in Figure 3, due to the larger energy imparted to the fluid flow. Not only does the deformation of the wedge becomes more severe for the higher fall, but it also varies significantly in shape. For the higher fall, the deformation of the panel seems to be characterized by higher vibration modes, which cause the curvature of the panel to change sign along its span.

While none of the panels failed for the shorter fall, Figure 3 shows that the VE220-60 panel failed between 7.5 and 12.5 ms close to the keel. The failure of the panel causes the onset of a flow recirculation in the vicinity of the keel, as shown in Figure 3c. The detachment of the panel from the holding frame forces the water to roll around it, likely forming a vortical structure which may be seen from the presence of the closed velocity contours near the keel in Figure 3c. By inspecting all the repetitions in condition Light-High, we confirm that the failure occurs between 9.5 and 10 ms after the wedge touches the water surface. All the panels in the trials of condition Light-High failed; the panel that did not fail is treated as an outlier and is omitted from all the analysis.

### 3.2. Analysis of the Wedge Motion

**Figure 4:**
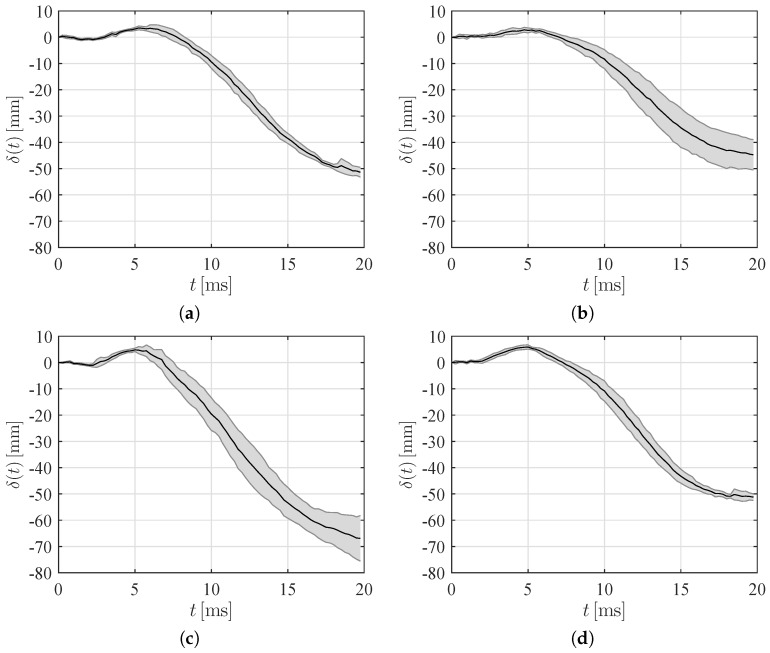
Time traces of the mean value of the tip deflection for (**a**) Light-Short; (**b**) Heavy-Short; (**c**) Light-High; and (**d**) Heavy-High conditions. The shaded area represents one standard deviation across repetitions.

To delve into the role of microballoon density and impact height, in Figure 4 we report the tip deflection for all the experimental conditions. Therein, we average data across the repetitions and compute a standard deviation. The tip deflection is calculated as $\delta \left(t\right)=-\frac{\xi_{k}\left(t\right)-\xi_{t}\left(t\right)}{\cos\beta }$, where $\xi_{k}$ and $\xi_{t}$ are the entry depth at the keel and the vertical displacement of the panel tip; see [37] for a detailed notation and an illustrative sketch. The sign of the tip deflection is positive when the panel bends downwards and negative when it bends upwards.

As expected, increasing the drop height produces an increase in both the upward and downward deflection for both the syntactic foams; see also Table 1. The effect of microballoon density on the upward deflection seems to be rather secondary, whereby the difference of the maximum tip deflection between the syntactic foams is within one standard deviation. With respect to the maximum downward deflection, we record a significant increase in the severity of the deformation for lighter syntactic foams; see also Table 1. This should be related to the larger compliance of the lighter panels, which results in a stronger hydroelastic coupling.

The time at which the peak is attained is marginally influenced by the microballoon density, but does not change with the impact height, as shown in Table 1. This suggests that the initial motion is primarily controlled by linear vibration, such that the magnitude of the deformation changes without altering the time scale of the process. In fact, the initial deformation of the panel is at most 1 cm, such that nonlinearities in the cantilevered panel of 11.5 cm are secondary. The slightly larger rise time recorded for the lighter panels may be explained by their higher compliance, which induces a slower vibration in the early stage of the impact.

**Figure 5:**
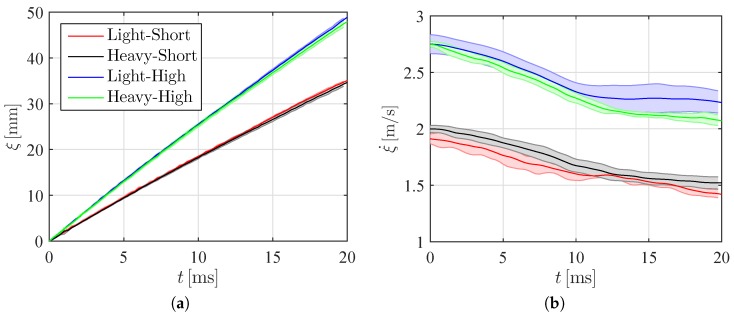
(**a**) Time trace of the mean entry depth and (**b**) mean velocity of the keel for different conditions. The shaded area represents one standard deviation across repetitions.

In Figure 5, we illustrate the time trace of the entry depth *ξ* and velocity $\dot{\xi }$ at the keel of the wedge for different experimental conditions. Similar to Figure 4, we average data across repetitions and compute a standard deviation. Results demonstrate that microballoon density had a minimal effect on the entry depth and velocity at the keel for the same impact height. Predictably, increasing the drop height causes a robust increase in the entry depth and speed. On the other hand, no noticeable differences are recorded for the two different microballoon densities in the entry depth or speed. Overall, the entry depth and speed do not seem to be influenced by the remarkable deformation of the wedge during the impact, even leading to failure for VE220-60 panels.

**Figure 6:**
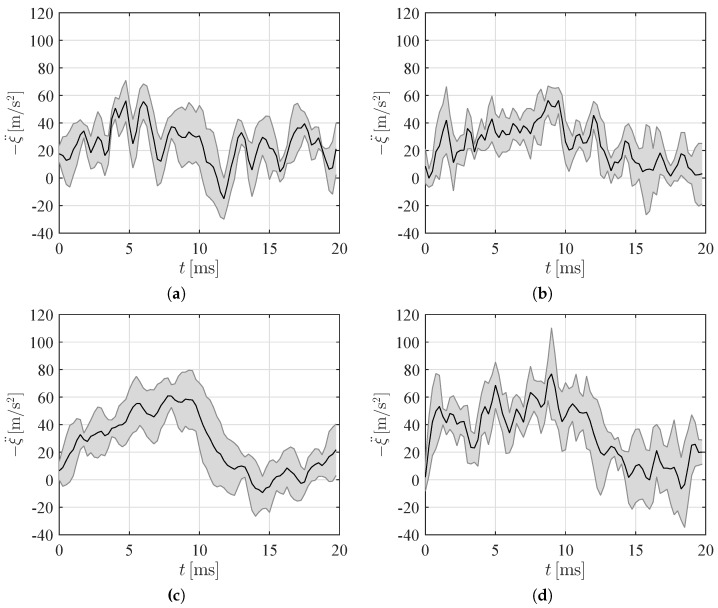
Time trace of the mean acceleration of the keel for (**a**) Light-Short; (**b**) Heavy-Short; (**c**) Light-High; and (**d**) Heavy-High conditions. The shaded area represents one standard deviation across repetitions.

To appreciate hydroelastic phenomena, we should resort to the study of the acceleration at the keel, displayed in Figure 6 for the different experimental conditions. For all the experimental conditions, we observ rapid oscillations in the acceleration, which should be ascribed to hydroelastic phenomena. In fact, for a rigid wedge in free-fall, the acceleration at the keel first reaches a positive peak and then decreases to zero as the wedge decelerateds during the water entry [35]. Similar to experiments on flexible metal and plastic structures [37,38,52], we observe that this trend is modulated by rapid oscillations at a frequency on the order of 1 kHz, associated with the vibration of the panels.

Figure 6a,b demonstrate that the acceleration signals for the 20 cm drop are comparable for the two types of syntactic foams; see also Table 1, where we report the maximum acceleration. Changing the impact height has a noticeable effect on the acceleration of the heaviest panels, which on average display a peak acceleration on the order of 10 g; see Figure 6c,d and Table 1. A similar increase is not observed for the lightest panel, which is likely due to hydroelastic phenomena that modulate the water entry. Although the lightest panels failed during the impact, the acceleration at the keel does not offer indications of the failure, except for a sudden decrease toward zero at the onset of the failure, which is however masked by experimental uncertainties and is perhaps only useful for a-posteriori analysis.

### 3.3. PIV-Based Reconstruction of the Pressure Field and Hydrodynamic Loading

**Figure 7:**
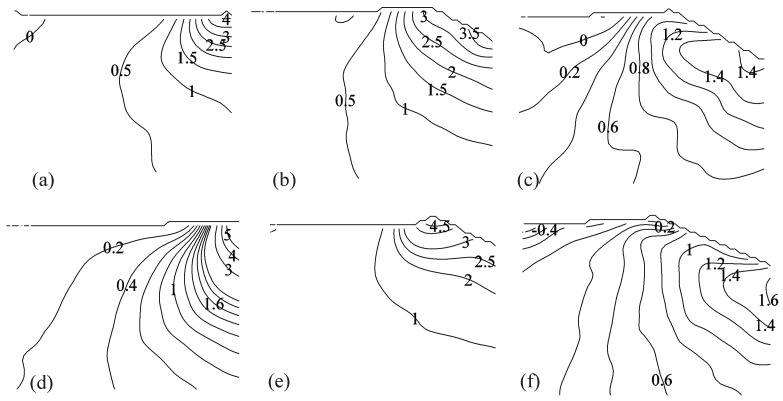
Pressure distribution in the fluid region in kPa for (**a**–**c**) Light-Short and (**d**–**f**) Heavy-Short conditions, at different time instants: (**a**,**d**) t=1.25 ms; (**b**,**e**) t=7.5 ms; and (**c**,**f**) t=12.5 ms.

**Figure 8:**
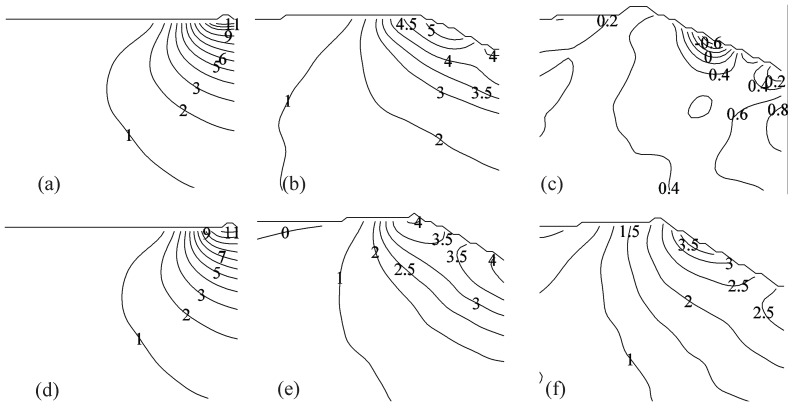
Pressure distribution in the fluid region in kPa for (**a**–**c**) Light-High and (**d**–**f**) Heavy-High conditions, at different time instants: (**a**,**d**) t=1.25 ms; (**b**,**e**) t=7.5 ms; and (**c**,**f**) t=12.5 ms.

Figure 7 and Figure 8 display PIV-based pressure reconstruction for the same time instants considered in Figure 2 and Figure 3. Results demonstrate that pressure is maximized in the early stage of the impact in the vicinity of the water jet for both the impact heights, consistent with previous findings on the water entry of rigid and flexible aluminum wedges [35,37]. As expected, the pressure field increases as the impact height increases, due to the concurrent increase in the magnitude of the velocity field.

Figure 8 demonstrates that the pressure field in the fluid domain is similar for both VE220-60 and VE460-60 panels in 40 cm fall at time instants t=1.25 and 7.5 ms, while the pressure in the fluid for the VE220-60 panel at t=12.5 ms is significantly different from the pressure field for the VE460-60 panel, due to the failure of the panel. Consistent with the velocity recirculation reported in Figure 3c, we find that the pressure reaches negative values in the vicinity of the failed panel, while being positive at the keel of the wedge.

**Figure 9:**
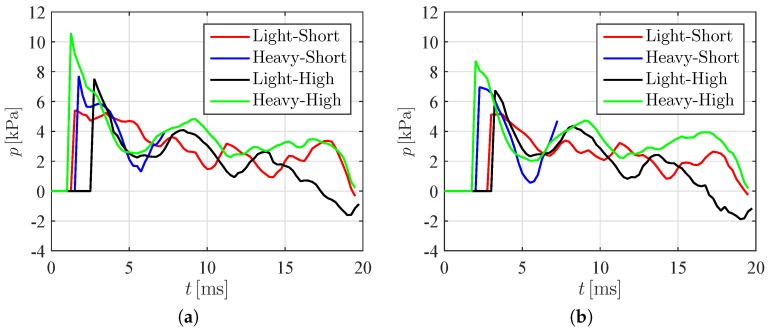
Hydrodynamic loading for different conditions at (**a**) 5 mm and (**b**) 10 mm away from the keel. Note that we only present results before failure for the VE220-60 panel falling from 40 cm.

To better clarify the hydrodynamic loading experienced by the panels during slamming, in Figure 9 we compare the pressure on the panels for two different distances from the keel. Figure 9 illustrates the hydrodynamic loading at 5 and 10 mm away from the keel for the same trials depicted in Figure 7 and Figure 8. For the VE220-60 panel falling from 40 cm, we only report the hydrodynamic loading before the panel failure.

As expected, the hydrodynamic loading increases by increasing the impact height from 20 to 40 cm. Figure 9 demonstrates that VE220-60 experiences a more modest hydrodynamic loading compared to VE460-60 for the same impact height. This counterintuitive finding should be attributed to hydroelastic effects, which could contribute to a diffusion of the hydrodynamic loading on the panel in the early stage of the impact. Similar evidence has been reported in Qin and Batra [53], Panciroli and Porfiri [37], and Shams and Porfiri [44] through the analysis of the so-called normalized pressure intensity factor, quantifying the intensification of the loading on the flexible wedge with respect to a rigid one. Results presented therein suggest the possibility of a beneficial effect of hydroelasticity in the early stage of the impact, which is gradually lost as the wedge penetrates the water surface.

### 3.4. Fracture Features

**Figure 10:**
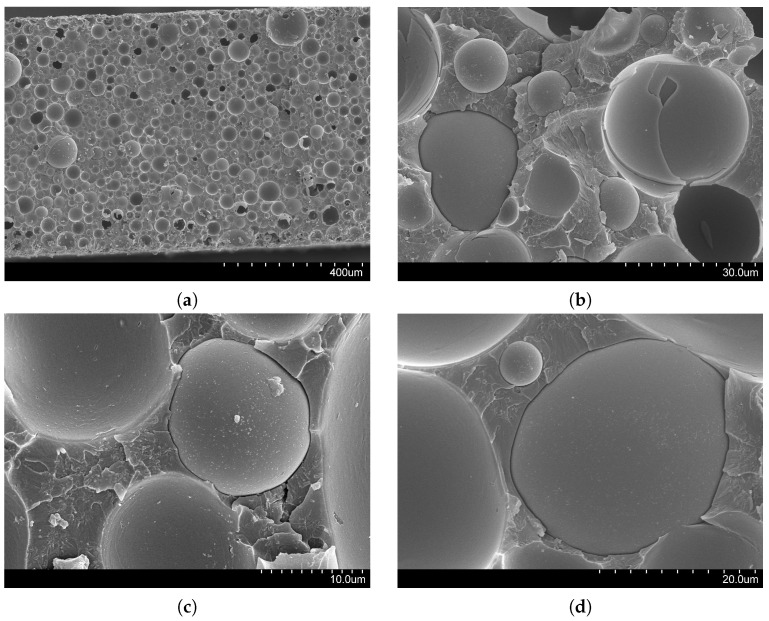
Fracture features of a failed VE220-60 panel: (**a**) Micrograph of the entire cross-section; (**b**) Magnified view of the top edge; (**c**) Magnified view of the bottom edge; and (**d**) Magnified view of the center.

Figure 10a reports the cross-section of the failed VE220-60 panel for the impact height of 40 cm, and in Figure 10b–d we present micrographs with higher magnification at different locations on the cross-section. In agreement with our expectations based on vinyl ester/glass syntactic foams [5,45], the micrographs suggest that failure is primarily influenced by debonding of microballoons from the matrix and crack growth in the matrix. Microballoon brittle fracture may only be noted at a few locations on the cross-section close to the top and bottom surfaces.

Specifically, Figure 10a does not show the presence of debris on the surface, thereby suggesting that failure is initiated by the failure of the panel on the tensile side of the cross-section during bending [5]. Different from a quasi-static three-point bending test [45], the tensile side of the cross-section alternates during the impact between the top and bottom edge, shown in Figure 10b,c. From the analysis of these images, we appreciate that microballoons could in fact debond from the matrix on both the top and bottom edges. As a result of debonding, most of the tensile stress induced by the water impact is resisted by the matrix, whose clearly-identifiable tensile cracks lead to the failure of the panel.

Some of the microballoons might also fracture during the water impact—especially those with pre-existing defects, as shown in Figure 10b. However, microballoon failure becomes less likely toward the center of the panel (as shown in Figure 10d), where matrix cracks initiated from the edges will ultimately propagate to determine the failure of the composite.

## 4. Conclusions

In this work, we investigated the water impact of vinyl ester/glass microballoons syntactic foams. Specifically, we analyzed the slamming response of syntactic foams with 60% inclusion volume fractions and two types of glass reinforcements, with nominal densities of 220 and 460 kg/m${}^{3}$. Thin syntactic foam panels were assembled in a miniature flexible wedge, impacting the water surface in a free-fall drop tower setup. Experiments were conducted for two different impact heights to explore the role of impact energy on syntactic foam response. High-speed imaging was used to measure the velocity of the fluid flow, the hydrodynamic loading, and the wedge deformation during the impact.

In agreement with our expectations, we found that the drop height has a primary role on the response of vinyl ester/glass syntactic foams, whereby both the panel deformation and the hydrodynamic loading increase. Less predictably, we also demonstrated a critical role played by the density of microballoons on syntactic foam response. We found that lighter microballoons could cause the failure of the panels during the impact, differently from heavier syntactic foams. This might be due the fluid–structure interaction, which causes lighter panels to experience a larger deformation during the impact. Such a larger deformation will ultimately translate into higher stress levels within the panel. As a result, the observed dependence on microballoon density is not in conflict with previous evidence in [10], where it was shown that microballoon density has a marginal role on unnotched Izod impact properties of vinyl ester/galss syntactic foams. In the present study, the impact response was the outcome of the complex interplay between the strength of the panels and their compliance, with only the latter determining the extent of hydroelastic coupling.

Overall, this study offers compelling evidence for the role of hydroelasticity on the slamming response of vinyl ester/glass syntactic foams. Future work should seek to establish predictive models for the response of syntactic foam panels to water impact, to further elucidate the mechanics of failure of syntactic foams. Current approaches for the study of water impact are largely based on linearized structural models [44,51], which cannot capture the severity of the deformations exhibited by the wedges considered in this experimental study. Once extended to tackle large deformations, these models could be coupled with available structural criteria for syntactic foam failure [45] to help explain the critical role of hydroelasticity.

## Figures and Tables

**Table 1:** Mean entry velocity $V_{0}$; maximum $\delta_{\max}^{+}$ and minimum $\delta_{\max}^{-}$ values of the tip deflection; $t_{\max}^{+}$ rise time to reach the maximum upward deflection $\delta_{\max}^{+}$; maximum value of the acceleration ${\ddot{\xi }}_{\max}$; and hydroelasticity factor $R_{F}$ along with standard deviations for the four experimental conditions. For the Light-High condition, the sample that did not fail was treated as an outlier and was not included in the computation of the mean and standard deviation for all the metrics listed below.

Condition	Syntactic Foam	*H* (cm)	$V_{0}$ (m/s)	$\delta_{\max}^{+}$ (mm)	$\delta_{\max}^{-}$ (mm)	$t_{\max}^{+}$ (ms)	$\left|{\ddot{\xi }}_{\max}\right|$ (m/s${}^{2}$)	$R_{F}$
Light-Short	VE220-60	20	1.911±0.046	3.96±0.64	−51.53±1.83	5.87±0.75	78.22±17.17	0.024
Heavy-Short	VE460-60	20	1.999±0.077	3.20±0.73	−44.89±5.82	5.29±0.64	72.36±5.90	0.029
Light-High	VE220-60	40	2.750±0.084	5.39±1.05	−67.18±8.36	6.00±0.69	83.83±7.81	0.016
Heavy-High	VE460-60	40	2.748±0.026	6.09±0.79	−51.67±1.29	5.25±0.14	98.48±15.99	0.021
